# COPII-dependent ER export in animal cells: adaptation and control for diverse cargo

**DOI:** 10.1007/s00418-018-1689-2

**Published:** 2018-06-18

**Authors:** Janine McCaughey, David J. Stephens

**Affiliations:** Cell Biology Laboratories, School of Biochemistry, University Walk, University of Bristol, Bristol, BS8 1TD UK

**Keywords:** COPII, Vesicle, Endoplasmic reticulum, Golgi, procollagen

## Abstract

The export of newly synthesized proteins from the endoplasmic reticulum is fundamental to the ongoing maintenance of cell and tissue structure and function. After co-translational translocation into the ER, proteins destined for downstream intracellular compartments or secretion from the cell are sorted and packaged into transport vesicles by the COPII coat protein complex. The fundamental discovery and characterization of the pathway has now been augmented by a greater understanding of the role of COPII in diverse aspects of cell function. We now have a deep understanding of how COPII contributes to the trafficking of diverse cargoes including extracellular matrix molecules, developmental signalling proteins, and key metabolic factors such as lipoproteins. Structural and functional studies have shown that the COPII coat is both highly flexible and subject to multiple modes of regulation. This has led to new discoveries defining roles of COPII in development, autophagy, and tissue organization. Many of these newly emerging features of the canonical COPII pathway are placed in a context of procollagen secretion because of the fundamental interest in how a coat complex that typically generates 80-nm transport vesicles can package a cargo reported to be over 300 nm. Here we review the current understanding of COPII and assess the current consensus on its role in packaging diverse cargo proteins.

## Introduction

Compartmentalization of cells is a common feature of all eukaryotes. This necessitates a transport system to traffic proteins between these compartments. The first membrane trafficking step for most newly synthesized membrane and secretory proteins is from the endoplasmic reticulum (ER) to the Golgi. The transport of proteins out of the ER is enabled by the coat protein complex type II (COPII) vesicles. Since our lab wrote a review on this topic in this journal in 2008 (Hughes and Stephens 2008) much has changed in terms of our understanding of the biology of COPII-dependent ER export, while some aspects have remained broadly similar. The fundamental underpinnings of the field reflect the incredible work of the early pioneers who defined the molecular basis of the membrane trafficking machinery (Barlowe et al. 1994; Schekman and Novick 2004) for which Schekman, Rothman, and Südhof were awarded the Nobel Prize for Physiology or Medicine in 2013. The detail with which the mechanism and regulation of the COPII machinery has been described, has increased considerably in recent years. The complexities of ER export in different organisms, during distinct stages of development, and in pathological conditions have become more of a focus (Zanetti et al. 2011).

**Fig. 1:**
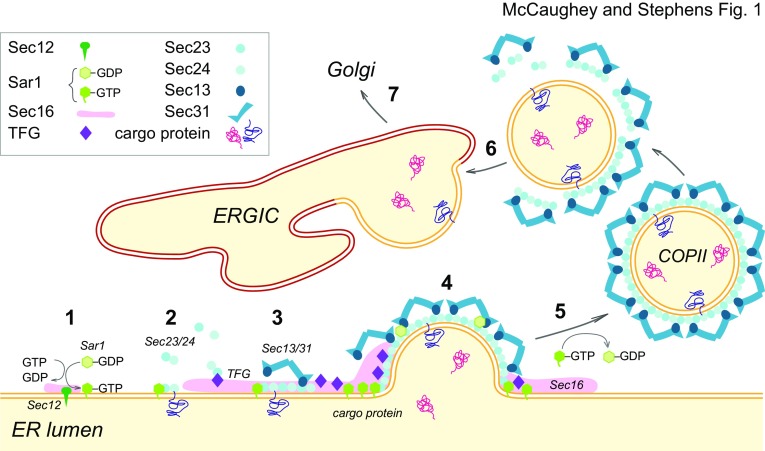
COPII-dependent packaging. (1) At ERES Sec16 binds the membrane protein Sec12, which acts as a GEF for Sar1–GDP promoting GDP–GTP exchange. Active Sar1–GTP then binds the ER membrane. (2) The inner COPII components Sec23/24 are recruited and Sec24 binds cargo proteins from the ER lumen. (3) TFG and Sec16 help organize the ERES to which Sec13/Sec31 are recruited by binding to the inner coat complex. (4) A COPII vesicle begins to form as the membrane deforms, Sar1–GTP accumulates at the base of this structure and undergoes conformational changes through GTP-hydrolysis induced by Sec23–Sec24 together with Sec13–Sec31. (5) The COPII vesicle containing the cargo proteins is released from the ER and traffics to the ERGIC. (6) Fusion with the ERGIC membrane takes place after uncoating of the vesicle. (7) Subsequently, cargo proteins are transported from the ERGIC to the Golgi

Our understanding of COPII vesicle formation remains centred around the core COPII components (Barlowe et al. 1994). Sec12 catalyses GDP–GTP exchange on Sar1 which in turn recruits Sec23–Sec24 to the ER. It is at this stage that cargo begins to accumulate and Sec13–Sec31 is recruited to complete the formation of the COPII coat and drive budding (Fig. 1). This minimal machinery, capable of reconstituting COPII coat assembly and membrane budding in vitro, was defined 20 years ago (Matsuoka et al. 1998) and drives efficient export of nearly all secretory cargo from the ER. The human genome encodes two isoforms of Sar1, two of Sec23, four of Sec24, and two of Sec31. These function in the same fundamental budding process but expand the possibilities for the machinery, not least to accommodate different cargo. What has changed in the last 20 years is our understanding of the additional complexity of the system in terms of spatial and temporal regulation.

The majority of the early work in this field came from yeast genetics and biochemical reconstitution. COPII is widespread throughout the eukaryotic branches of the tree of life. The conservation of the core machinery between species is best illustrated by the fact that Sec12, Sec16, Sar1, Sec13, Sec31, Sec23, and Sec24 were all present in the Last Eukaryotic Common Ancestor [LECA, (Schlacht and Dacks 2015)]. In plants the fundamentals of COPII-dependent budding are highly conserved. The specializations that occur to adapt the process arise from both the different organization of the ER–Golgi interface in plants (Hawes et al. 2010) and the issues faced in building and maintaining plant cells. In both plant and animal cells, multiple isoforms of the core COPII components exist and many have been shown to undertake specialized tasks (Chung et al. 2016; Zeng et al. 2015). Notably, multiple isoforms of Sec24 are identifiable even in the LECA which has evolutionary implications for early complexity within this system and has relevance to the membrane trafficking systems which use similar modular structures. This review will focus on COPII in animal cells.

## Mechanisms of protein sorting

Sec24 is the major cargo binding module within the COPII coat (Miller et al. 2002). We now have an atomic level understanding of many cargo recognition events thanks to X-ray crystallography and cryo-electron microscopy (EM). This, coupled with other functional studies, has revealed an enormous diversity in the cargo recognition capabilities of Sec24. Multiple cargo binding sites have been defined on the surface of Sec24 (Miller et al. 2002, 2003) that ensure selective capture of diverse cargo bearing specific amino acid motifs or adopting specific conformations. The ability to recognize diverse cargo is further expanded by the presence of multiple COPII isoforms. This diversity of Sec24 isoforms is clearly important as well illustrated by the functional sorting of SNARE complexes (Adolf et al. 2016)—different isoforms of Sec24 can recruit distinct fusogenic SNAREs to physically separate the fusion factors and thus prime vesicles for subsequent homotypic fusion. Other components of the COPII coat also contribute to cargo sorting, for example, mouse genetics suggests that individual Sec23 isoforms might sort distinct cargo (Zhu et al. 2015). Furthermore, some cargoes are sorted by receptors which recognize their transmembrane domains, e.g. Erv14p (Herzig et al. 2012). Receptors can act in combination with other sorting motifs to ensure the fidelity of export through coincidence detection (Pagant et al. 2015). Cargo sorting by COPII can also be dependent on oligomerization (Springer et al. 2014) or multimeric sorting signals (Nie et al. 2018). The role of COPII, acting in combination with other quality control systems to ensure the selectivity of cargo export, has been discussed extensively elsewhere (Barlowe and Helenius 2016).

Disease-linked mutations in COPII often link to defects in packaging of specific cargo proteins. This is likely explained by the essential nature of the COPII system, meaning that severely deleterious mutations are not compatible with life. Those mutations that alter the packaging of specific proteins, on the other hand, can be tolerated and demonstrate the specificity of interaction between different COPII isoforms and cargo. Mutations in Sec23A that cause cranio-lenticulo-sutural dysplasia are a prime example of this (Boyadjiev et al. 2006). Here, inefficient assembly of COPII leads to defects in packaging of procollagen (Bi et al. 2007; Fromme et al. 2007). Similarly, there is an essential requirement for Sec24B in the trafficking of the planar cell polarity protein Vangl2; mutations in Sec24B in mice cause craniorachischisis (Merte et al. 2010; Wansleeben et al. 2010) and related mutations in humans result in neural tube defects (Yang et al. 2013). Mutations in Sar1B are known to cause chylomicron retention diseases including Anderson’s disease (Jones et al. 2003). This is considered to be a result of alterations in the flexibility of the COPII cage (Miller and Schekman 2013; Zanetti et al. 2013).

Other diseases have less clear links to cargo packaging. Notably, congenital dyserythropoietic anaemia type II (CDA II) is caused by mutations in Sec23B (Bianchi et al. 2009; Schwarz et al. 2009). This might be underpinned by broad defects in glycosylation, but no specific cargo packaging defect has been identified. Further work in this field has been somewhat hampered by fundamental differences between humans and mice with regard to genotype–phenotype correlation (Khoriaty et al. 2014; Satchwell et al. 2013). These studies highlight the importance of understanding subunit diversity. In zebrafish, knockout of either Sec23A or Sec23B leads to craniofacial development defects showing that they are not functionally redundant (Lang et al. 2006). In humans, mutations in Sec23B cause CDAII (Bianchi et al. 2009; Schwarz et al. 2009), but this is not recapitulated in mouse knockout models (Khoriaty et al. 2014). This is likely due to expression of Sec23A in the later stages of erythroid differentiation in mice but not humans (Satchwell et al. 2013). Thus, the roles of individual components need to be considered carefully, particularly where multiple isoforms and splice forms are concerned.

## Organization of ER exit sites

In animal cells the COPII budding process is organized at discrete sites on the ER membrane termed the transitional ER (Bannykh et al. 1996). This region of the ER is defined by Sec16 which is tightly associated with the ER membrane (Hughes et al. 2009; Watson et al. 2006). This directs the formation of COPII-coated structures that contain Sec23–Sec24 and Sec13–Sec31. Collectively, these sites of budding and the COPII-coated structures that emerge from them are called ER exit sites (ERES). In our definition (Hughes et al. 2009; Watson et al. 2006), the entire “ER exit site” includes the transitional ER (Mezzacasa and Helenius 2002; Soderholm et al. 2004), defined by the presence of Sec16 (Connerly et al. 2005; Hughes et al. 2009), the canonical COPII coat and COPII-coated membranes (Bannykh and Balch 1997), and membranes of the ER–Golgi intermediate compartment (ERGIC). Therefore, ERES are the sites at which COPII vesicles form and transfer cargo to the ERGIC. The ERGIC remains an enigmatic organelle, seeming to act as an intermediate sorting and processing station between the ER and Golgi, perhaps with a role in maintaining the compartment identity of each organelle (Appenzeller-Herzog and Hauri 2006). As we will see later, there is also evidence that the ERGIC, under some circumstances, has a direct role in the formation of COPII-dependent carriers.

Several key components have been identified that appear to play central roles in defining and organizing ERES in animal cells. Sec16 is present in yeast and early work showed how it potentiates COPII vesicle formation. In animal cells, Sec16 appears to both organize and regulate (Bharucha et al. 2013) the COPII budding process (Yorimitsu and Sato 2012). Its central domain, which has Sec13 embedded within it (Whittle and Schwartz 2010), directs tight association with the ER membrane, while the C-terminal domain binds to several other subunits of the COPII complex. The guanine nucleotide exchange factor Sec12, binds to Sec16 at ERES (Montegna et al. 2012) to initiate the exchange of GDP for GTP on Sar1 and to concentrate Sar1–GTP at these sites (Montegna et al. 2012) thus driving the initial stages of coat formation (Fig. 1, steps 1–4). Sec16 mutations have been defined in yeast that impact on the Sar1-GTPase cycle (Kung et al. 2012). Sar1–GTP hydrolysis, triggered by Sec23 together with Sec13 (Antonny et al. 2001; Yoshihisa et al. 1993), is followed by vesicle fission (Fig. 1, step 5). COPII vesicles undergo uncoating prior to merging with the ERGIC (Fig. 1, step 6), before being transported further to the Golgi (Fig. 1, step 7).

A major step in our understanding of the physical nature of ERES sites came from the identification of TFG (Trk-fused gene). Originally named as a gene product fused to the Trk receptor, TFG has since been defined as a key component of the ER exit site (Johnson et al. 2015). TFG interacts with Sec23, i.e. the inner layer of the COPII coat (Hanna et al. 2017) as well as Sec16 (Witte et al. 2011). Binding of TFG to Sec23 occurs through an interface on Sec23 that is shared with both the outer COPII coat (Sec13–Sec31) and the cargo receptor, TANGO1 [Transport and Golgi Organization 1, (Hanna et al. 2017)]. TANGO1 is unusual among cargo receptors in that it is not incorporated into the final vesicle (Saito et al. 2009). We will discuss the role of TANGO1 later. The current model is that TFG tethers partially uncoated vesicles to retain them in close proximity to the ERGIC (Hanna et al. 2018, 2017). Our own work has shown that TFG acts to organize the ERES into a larger structure and that this might facilitate the export of larger cargoes (McCaughey et al. 2016). An alternative model here is that TFG ensures the close apposition of ERGIC membranes with the ER to facilitate nascent bud expansion and efficient transfer of procollagen to the ERGIC and on to the Golgi. The role of TFG has been reviewed recently and readers are referred to that article for more depth and detail on this important protein (Hanna et al. 2018). In many ways, one can consider TFG an integral component of the COPII budding machinery in animal cells.

Other work has shown that TANGO1 has a role in ERES organization (Maeda et al. 2017) independent of its role in cargo binding (Rios-Barrera et al. 2017). Another cargo receptor, SURF4 (SFT-4 in *C. elegans*, Erv29p in yeast), involved in the export of lipoproteins from the ER, has recently been shown to be involved in ERES structure (Saegusa et al. 2018). Overall, there is considerable evidence that perturbing the normal cycle of COPII budding has a direct impact on the overall structure of the ERES, thus, ERES structure and function appear inextricably linked. Many other factors have also been identified that modulate, optimize or regulate the process.

## Efficient assembly of the COPII coat as a prerequisite for procollagen transport

**Fig. 2:**
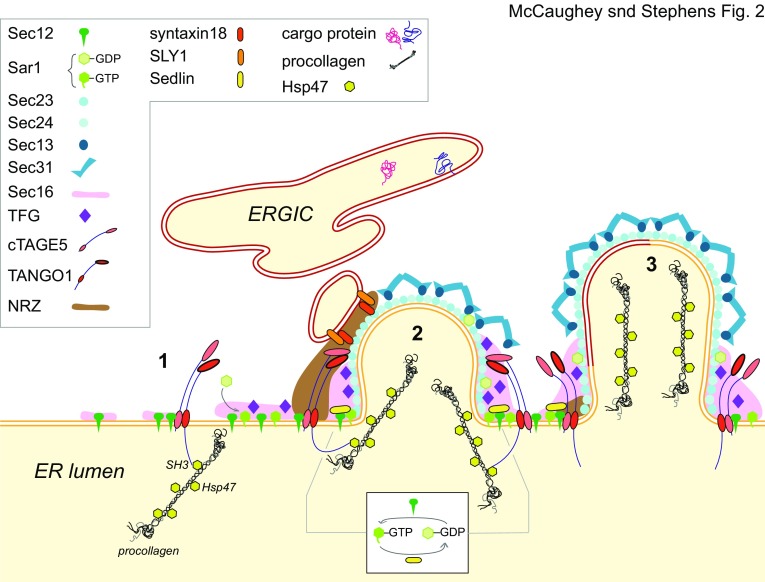
Proposed role of TANGO1 and cTAGE5. (1) cTAGE5 binds Sec12 and concentrates it to the ERES. TANGO1 binds Hsp47 through its ER-luminal SH3-domain, directing procollagen to ERES. (2) Efficient Sar1–GTP/GDP cycling is facilitated by sedlin and Sec12. The COPII prebudding complex is further regulated by TANGO1 together with cTAGE5 that bind to the COPII inner layer. The NRZ tethering complex is recruited by TANGO1/cTAGE5 and recruits ERGIC membranes to the forming COPII complex. TANGO1/cTAGE5 assemble the fusion machinery SLY1 and syntaxin18 that drive incorporation of ERGIC membranes into the vesicle to allow procollagen incorporation into the carrier (3). Some uncertainty remains as to the precise structure of procollagen trimers on exit from the ER such that larger COPII-coated carriers might not be necessary

Originally identified as a gene required for general protein secretion in *Drosophila* (Bard et al. 2006), TANGO1 has since been shown to act as cargo receptor for procollagen VII in vertebrates (Saito et al. 2009). This definition has more recently been extended to other procollagens (Wilson et al. 2011) and other unusually large cargo (Rios-Barrera et al. 2017) including lipoproteins (Santos et al. 2016). Procollagen is the most abundant protein in the human body and large fibrillar procollagens can form trimers of > 300 nm potentially while within the ER (Bachinger et al. 1982). These trimers must then be exported, presenting a challenge to the COPII machinery which classically generates 60–90 nm vesicles. This has led to considerable work defining the role of TANGO1 in the COPII cycle. Its function is inextricably linked with that of cTAGE5 [cutaneous T-cell lymphoma-associated antigen 5, Saito et al. (2011)] and other related proteins including TALI (Santos et al. 2016). Intriguingly, TANGO1 appears to control some of the quite fundamental aspects of the COPII budding event, notably the localization of Sec12 through binding to cTAGE5 [(Saito et al. 2014b), Fig. 2, step 1]. Indeed, its original identification was from a screen using horseradish peroxidase as a secretory reporter, suggesting a general role in secretion more broadly (Bard et al. 2006). In addition, a shorter form of TANGO1 (TANGO1S), lacking the procollagen binding domain, exists and both TANGO1L and TANGO1S are equally required for procollagen secretion (Maeda et al. 2016). It is difficult to reconcile these data with a dedicated role in the transport of large cargo. That said, some further support for a direct role of TANGO1 in procollagen export comes from experiments looking at the procollagen-specific chaperone Hsp47 (Ito and Nagata 2017; Nagata et al. 1986). TANGO1 binds directly to Hsp47 (Ishikawa et al. 2016). Hsp47 interacts with the SH3 domain of TANGO1 and can bind to both monomeric (Hosokawa and Nagata 2000) and trimeric procollagens, with a higher affinity for the latter (Fig. 2, step 1), thus acting as a cargo adapter with no specificity for particular procollagen types (Ito and Nagata 2017; Koide et al. 1999; Tasab et al. 2000).

TANGO1 assembles a fusion machinery that includes Sly1 and syntaxin 18 to drive heterotypic fusion between COPII vesicles and ERGIC membranes [(Nogueira et al. 2014), Fig. 2, step 2]. This provides a mechanism by which close apposition of COPII-coated buds and the ERGIC would provide additional membrane to expand the nascent COPII-derived carrier. This is considered to solve the issue of carrier expansion to encapsulate large cargoes including lipoproteins and procollagens (Fig. 2, step 3). This model has since been further developed to show that TANGO1 recruits the NRZ tethering complex that includes NBAS, RINT1, and ZW10 [(Raote et al. 2018) Fig. 2, step 2]. This mode of action is inextricably linked to the formation of rings of TANGO1 at ERES (Liu et al. 2017; Raote et al. 2017). NBAS mutations in humans cause multisystem disorders (Segarra et al. 2015) that do include defects in bone formation (Balasubramanian et al. 2017) and can therefore be linked to impaired procollagen transport.

One can consider that procollagen and other large cargoes such as lipoproteins present an atypical problem to the COPII system. It remains unclear just how much of a problem. Indeed, recent statistical analysis of the structure of procollagen obtained from atomic force microscopy shows that the procollagen trimer is more flexible than has been considered previously (Rezaei et al. 2018). The persistence length of procollagen trimers exiting the ER remains undefined. The typical figure of 300 nm for fibrillar type I collagen comes from experiments using purified collagen from extracellular matrix (Bachinger et al. 1982). It is therefore possible that the “rod”, which has not yet acquired all its post-translational modifications, could be capable of bending to be accommodated into smaller carriers or that other mechanisms are in operation. As such we consider that the secretion of procollagen requires an optimal COPII system and indeed an optimal secretory pathway. This explains why perturbation of many COPII components, e.g. Sec23A (Boyadjiev et al. 2006; Cox et al. 2018; Lang et al. 2006), Sec23B (Lang et al. 2006), Sec24D (Garbes et al. 2015; Ohisa et al. 2010), Sec13 (Townley et al. 2008, 2012), as well as global regulators of COPII expression such as the transcription factor CREB3L2 (Melville et al. 2011), all result in obvious defects in procollagen secretion and extracellular matrix assembly. The same can be seen when one considers key trafficking machineries in the ER–Golgi pathway such as the TRAPP tethering complex protein Sedlin [(Venditti et al. 2012) Fig. 2, step 2], and the golgins giantin (Katayama et al. 2011) and GMAP-210 (Smits et al. 2010). For lipoproteins, the actual site at which extensive neutral lipid is added to nascent lipoproteins forming at or adjacent to the ER is also ill-defined leaving open to question the size of lipoproteins at the point of ER exit. Thus, there are possible alternatives to the idea of a dedicated “large-cargo” machinery. Regardless, the weight of evidence supports full the notion that procollagen and lipoproteins are exported from the ER in a COPII-dependent manner.

## Flexibility of the COPII coat: the case for large COPII-dependent carriers

**Fig. 3:**
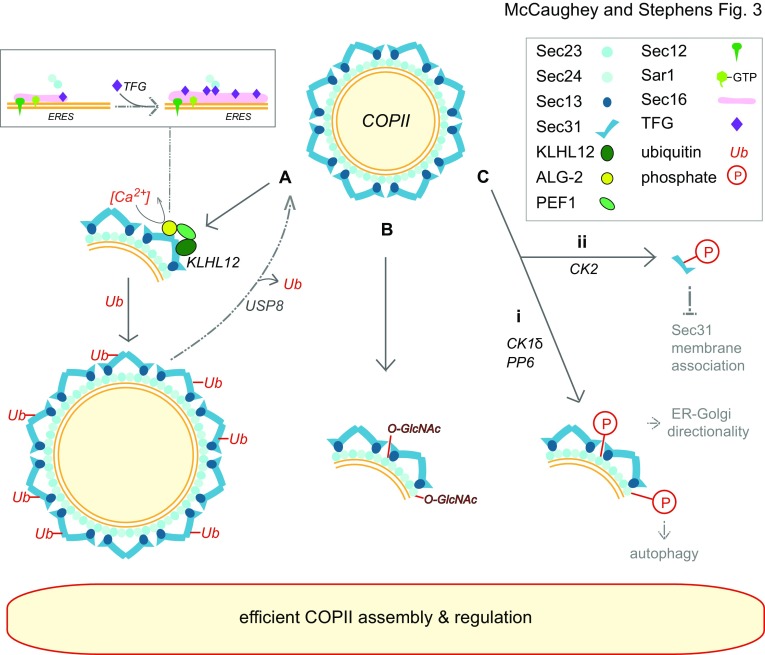
Post-translational modification of the COPII coat for efficient coat assembly. COPII vesicles can be modified in several ways. (A) Monoubiquitylation of Sec31A is facilitated by the CUL3-KLHL12 ubiquitin ligase in a calcium-dependent manner and helps regulate COPII size. This process can be reversed by USP8. ALG-2, a subunit of the KLHL12 complex, facilitates enrichment and assembly of TFG at ERES. (B) O-glycosylation through addition of *O*-*N*-acetylglucosamine to Sec24 and Sec23 is important for organization and regulation of the COPII complex. (Ci) Phosphorylation of Sec23/24 through CK1δ and PP6 confers directionality on COPII vesicles from ER to Golgi, while phosphorylation by CK2. (Cii) Inhibits association of Sec31 with the membrane. Sec24 phosphorylation is also involved in autophagy. All of these processes are necessary for efficient COPII assembly

Much of the debate around the nature of COPII carriers is linked to the inherent flexibility of the coat. In terms of the mechanics of ER export one cannot underestimate the contribution of bilayer composition. The importance of COPII in overcoming membrane tension was nicely shown in yeast (Copic et al. 2012). Mutants that result in increased membrane flexibility can bypass a requirement for Sec13 in the COPII budding process. These data strongly support models where the efficiency of the COPII-dependent budding machinery is critical to its normal function. While these data (Copic et al. 2012) implicate Sec13 in imparting a rigidity to the outer layer of the coat, in vitro the coat can generate a diverse array of shapes and sizes (Russell and Stagg 2010) including more tubular structures (Bacia et al. 2011). Much of this flexibility arises from the hinge region at the end of the major α-solenoid domains of the outer layer Sec13–Sec31 module (Noble et al. 2013). These data suggest that COPII may indeed be capable of forming large carriers able to encapsulate procollagen and other large cargoes. Evidence for the formation of large COPII carriers within cells is provided by work studying the ubiquitin ligase adaptor KLHL12 as a key modifier of the COPII budding event (Jin et al. 2012). KLHL12 directs monoubiquitylation of Sec31 which is reportedly required for the formation of large flexible procollagen-containing carriers (Fig. 3A). Deubiquitylation by USP8 antagonizes this step (Kawaguchi et al. 2018). The data showing that this ubiquitylation step modulates the secretion of procollagen are extensive. Whether that occurs in larger COPII-coated, or COPII-derived, structures is perhaps less clear.

Several publications (Gorur et al. 2017; Jin et al. 2012; McGourty et al. 2016) have defined large COPII positive structures which can be induced by KLHL12 overexpression and these occasionally co-label for procollagen. Some of the evidence that these are bona fide long-range carriers of procollagen does remain equivocal. In the original work, overexpression of FLAG-KLHL12 was shown to generate large structures (> 300 nm) that also labelled for Sec31A (Jin et al. 2012). The structures are few in number and COPII labelling appears very different to that one might expect from many other studies (Jin et al. 2012).

Subsequent work extended the mechanism of KLHL12-mediated ubiquitylation showing that PEF1 and ALG-2 are both subunits of the CUL3-KLHL12 ubiquitin ligase that modifies Sec31A (McGourty et al. 2016). ALG-2 has long been known to be a calcium-regulated modulator of COPII (Shibata et al. 2007; Yamasaki et al. 2006) and this new study introduced the concept of calcium-dependent regulation of COPII ubiquitylation. Through modulating COPII kinetics (Helm et al. 2014; la Cour et al. 2013; Shibata et al. 2010) ALG-2 primarily acts to stabilize the nascent coat and is thus considered a prerequisite for large vesicle formation. PEF1 was also shown to modulate COPII kinetics in yeast (Yoshibori et al. 2012). Intriguingly, ALG-2 is required for the localization and assembly of TFG [(Kanadome et al. 2017), Fig. 3A]. There is also evidence that PEF1 and ALG-2 together control transport of smaller cargoes such as the temperature sensitive viral glycoprotein VSV-G (Rayl et al. 2016). VSV-G has been used for many years as a reporter of biosynthetic membrane trafficking (Gallione and Rose 1985) and is widely considered a marker of canonical ER-to-Golgi-to-plasma membrane transport. As with the earlier work, here a small number of large structures were seen in cells overexpressing KLHL12 that labelled for Sec31A, PEF1 and ALG-2 (McGourty et al. 2016). This work also showed for the first time that these larger structures can be labelled with antibodies against procollagen and that depletion of either ALG-2 or PEF1 inhibited secretion of procollagen in HT1080 cells overexpressing type I procollagen (McGourty et al. 2016). Then, in 2017, the Schekman group reported large, procollagen- and Sec31A-positive structures in KI6 cells (Gorur et al. 2017). It is important to note that these KI6 cells overexpress both type I procollagen and FLAG-KLHL12. Super-resolution imaging showed that these observed structures are vesicular-like and with a “hollow” core. Correlative light electron microscopy showed apparent encapsulation of procollagen by large Sec31A puncta while smaller Sec31A puncta never colocalized with procollagen (Gorur et al. 2017). Live cell imaging using procollagen-GFP also showed short-range movement of these carriers over distances of a few microns. Further support for this model came from in vitro budding experiments showing COPII-dependent packaging of procollagen into large structures (Gorur et al. 2017; Yuan et al. 2017).

While these data are substantial and support a model in which procollagen traffics in large COPII carriers, we still retain some reservations about the large-vesicle model as it stands. As with previous work, we noted in these data the small number of Sec31A-positive puncta evident in many of the images (Gorur et al. 2017; Jin et al. 2012; McGourty et al. 2016). The motile structures observed (Gorur et al. 2017) are not shown to undergo long-range transport. The time-lapse sequences shown contrast to those from our original work showing procollagen-GFP translocating in small puncta over long-range curvilinear tracks (Stephens and Pepperkok 2002). These larger structures are only observed in cells overexpressing KLHL12 and are not entirely consistent with labelling of endogenous procollagen in many cell types. COPII labelling is often quite atypical in these cells and such large well-defined carriers have not been seen in work using cells that secrete high levels of procollagen including fibroblasts or epithelial cells. This includes IMR-90 fibroblasts shown in the supplemental data of Gorur et al. (2017). More compelling data comes from in vitro budding assays, but even here few examples are shown.

Overall, the weight of evidence in favour of roles for TANGO1, KLHL12, and the ubiquitin machinery acting with COPII in the transport of procollagen from the ER is substantial. The role of these proteins in procollagen export does not require the formation of larger vesicles and therefore is not incompatible with other models. Here, we merely wish to highlight the idea that large vesicles might not be either the only or the whole story.

## Additional modulators of the COPII system

Several other modifiers of COPII assembly have also been identified, but their mode of action is less well defined. The phospholipase-like protein p125 binds to multiple COPII components. Originally identified as a Sec23-interacting protein [Sec23IP, (Tani et al. 1999)], it acts to stabilize the interaction of the outer and inner layers (Ong et al. 2010). It contains a lipid binding domain that is key to its function in overall COPII-dependent ERES function (Klinkenberg et al. 2014; Shimoi et al. 2005). Knockout of p125/Sec23IP causes defects in spermiogenesis (Arimitsu et al. 2011), but how this is linked to COPII function remains unclear. Phenotypes are likely complicated here by other p125 family members (Nakajima et al. 2002).

Alternative splicing of Sec16 has been described (Wilhelmi et al. 2016), e.g. of exons encoding regions at the C-terminus of Sec16 which were found during T-cell activation. This appears linked to the efficiency of COPII activity which is greatly enhanced on T-cell activation. Notably, exon 29 encodes a region that interacts with Sec23 (Wilhelmi et al. 2016). This mechanism could therefore provide opportunities to fine-tune the efficiency of COPII-dependent budding at a cellular level. Many other COPII subunits also exist as multiple splice forms providing greater scope for diversity than can arise from the multiple paralogues of each subunit. The tissue- or stage-specific relevance of this remains ill defined.

Further modulation of COPII comes from transcriptional control. CREB3L2/BBF2H7 knockout mice display sever chondrodysplasia (Saito et al. 2009). In fibroblasts from these animals the ER is distended and collagen II and another matrix molecule, COMP, are retained. The zebrafish *feelgood* mutation is also caused by disruption of the CREB3L2/BBF2H7 gene (Melville et al. 2011). This mutant shares many phenotypic features with *crusher* fish which have a mutation in Sec23A. Indeed, the first transcriptional target identified for CREB3L2/BBF2H7 in this context was Sec23A. Subsequent work showed that CREB3L2/BBF2H7 regulates the expression of genes encoding a subset of COPII proteins: Sec23a, Sec23b and Sec24d (but not Sec24C), as well as TANGO1 and KLHL12 (Ishikawa et al. 2017; Melville et al. 2011). CREB3L2/BBF2H7 is responsive to ER stress, during which it is cleaved into an N-terminal domain that regulates transcription directly and a C-terminal domain that is secreted and acts on neighbouring cells to promote proliferation via hedgehog signalling (Saito et al. 2014a). This transcriptional system is also responsive to IGF-I (Ishikura-Kinoshita et al. 2012). Such insights into the physiological regulation of COPII-dependent trafficking offer some potential in terms of regenerative medicine and addressing age-related degeneration of tissues.

## Post-translational modification of COPII

COPII vesicle budding is a constitutive process continuously supplying newly synthesized proteins to the endomembrane system and extracellular space. However, the COPII machinery is also subject to complex regulation by post-translational modification. Cytosolic O-glycosylation of Sec24, through addition of *O*-*N*-acetylglucosamine (O-GlcNAc), has been shown to occur in interphase [(Dudognon et al. 2004), Fig. 3B]. This was originally reported to act antagonistically to phosphorylation to control Sec24 activity or interactions during cell cycle progression. Phosphorylation, however, plays a much wider role in the regulation of COPII as we will discuss later. More recent work has shown that, as with ubiquitylation (Jin et al. 2012; McGourty et al. 2016), the formation of a collagen rich extracellular matrix also appears to be regulated by O-GlcNAcylation of COPII proteins (Cox et al. 2018). As discussed above, knockout of Sec23A results in intracellular retention of procollagen. In this work (Cox et al. 2018), expression of a form of Sec23A that could not be O-GlcNAc modified failed to restore normal procollagen trafficking in the null background. Of note, perturbation of O-GlcNAc modification of Sec23A also perturbed canonical trafficking of VSV-G and decreased the overall pool of membrane associated Sec23A and Sec31A (Cox et al. 2018).

Further work has identified mechanisms that control key aspects of the COPII-dependent trafficking, such as directionality (to ensure directional flow of cargo to the Golgi rather than its return to the ER). The Golgi-localized protein kinase CK1δ (Lord et al. 2011) and the protein phosphatase PP6 (Bhandari et al. 2013) both act on Sec23–Sec24 (Fig. 3Ci). This is considered to drive directionality in the system by directing a hierarchy of protein–protein interactions from within the inner layer of the COPII coat through to the budding and subsequent tethering and fusion machinery (Lord et al. 2013). Other protein kinases can also modify COPII including CK2 which inhibits the association of Sec31 with the membrane [(Koreishi et al. 2013), Fig. 3Cii] as does the G1 cyclin-dependent kinase CRK1, at least in trypanosomes (Hu et al. 2016). Many of the other experiments highlighting roles for phosphorylation of COPII, particularly of Sec24, point to roles in autophagy.

## COPII and proteostasis: autophagy and the UPR

The balance between protein folding and ER export is an under-developed area of the COPII field. There is substantial evidence of an important and direct role for COPII in multiple aspects of ER quality control. Notably, an atypical role for COPII has been described in segregating misfolded proteins within the ER (Kakoi et al. 2013). Reciprocally, ER stress can regulate the COPII cycle (Amodio et al. 2013). In *Drosophila*, mechanisms have been described that operate to protect the core COPII machinery at times of cellular stress such as amino acid starvation (Zacharogianni et al. 2014). Nutrient deprivation of course also links directly to the field of autophagy. Here, there has been considerable activity in recent years showing key roles for COPII in phagophore initiation (Wang et al. 2014), LC3 lipidation (Ge et al. 2013, 2014) and autophagosome membrane expansion (Ge et al. 2017; Lemus et al. 2016). COPII is subject to control by phosphorylation during these events including modification of Sec23 (Gan et al. 2017) and Sec24 (Davis et al. 2016). This has been reviewed in more detail elsewhere (Davis et al. 2017). A further possible point of integration between COPII and autophagy is Tectonin β-propeller containing protein 2 [TECPR2, (Stadel et al. 2015)]. TECPR2 appears to regulate the levels of Sec24 in cells and, as such, ERES function. Confusingly it controls ER export through a mechanism dependent on its binding to lipidated LC3C. Both TECPR2 and LC3C are required for autophagosome biogenesis suggesting that this might be integrated via ERES function.

## Future perspectives

As with most areas of classical molecular cell biology much remains to be discovered about the COPII system. How it is adapted in different tissue contexts, in different organisms, and at different stages of development (Ishikawa et al. 2017) is clearly important to understanding its fundamental biology. The emerging roles for COPII during infection and during diverse disease pathologies underpin the need to understand this system in detail. The fundamental nature of the process also provides opportunities in industrial biotechnology, notably to produce secreted biologics and therapeutic antibodies. The possibility to modify the system for enhanced production holds some promise for these industries. Advancements in our understanding will also undoubtedly come from new developments in technology. Cryo-electron microscopy has also shown much promise here, both for COPII (Zanetti et al. 2013) and other vesicle systems (Dodonova et al. 2017; Halebian et al. 2017). The analysis of intact vesicles containing cargo (and unusual cargo) using proteomic profiling can be used to build on existing work in these areas (Margulis et al. 2016). Similarly, mass spectrometry enables us to understand the structural biology of COPII in more depth (Noble et al. 2013). Further advances in our ability to reconstitute these processes in vitro (Iwasaki et al. 2017; Yuan et al. 2017) will also extend our understanding of spatial and temporal control of COPII assembly and disassembly.

COPII has defined roles in viral replication including the turnip mosaic virus (Jiang et al. 2015), brome mosaic virus (Li et al. 2016), poliovirus (Trahey et al. 2012), and foot and mouth disease virus (Midgley et al. 2013). It also facilitates the life cycles of some key pathogens including *Salmonella* (Santos et al. 2015), *Listeria* (Gianfelice et al. 2015), enteropathogenic *E. coli*, and *Citrobacter* (Thanabalasuriar et al. 2013). Mutations in some COPII genes are implicated in osteogenesis imperfecta and related disorders (Garbes et al. 2015), as well as other diseases including cancer (Lee et al. 2016; Yehia et al. 2015). The extensive cross-talk between canonical COPII budding and metabolic pathways is clear. The interface with autophagy is one example as is the regulation of plasma cholesterol levels (Chen et al. 2013).

The role of core membrane trafficking pathways in neuronal function remains a relatively underdeveloped field. Dendritic development requires the COPII pathway (Ye et al. 2007). The presence of widespread dendritic ERES which are clearly used extensively (Evans et al. 2017), coupled with the presence of Golgi outposts, provides opportunities for local synthesis and secretion. Whether these pathways have relevance in neuronal degeneration, either in normal aging or disease, is not clear.

Considerable advances in cell biology have been made using small molecule inhibitors. There is some scope here for further development and some early work in this area has already identified potential inhibitors of COPII-dependent budding (Yonemura et al. 2016). The essential nature of the COPII pathway means that specific inhibitors are unlikely to be of therapeutic benefit; however, there might be further scope in targeting cargo-specific interactions, the interplay between protein folding and export, or the efficiency of the process itself.

Our core understanding is also likely to be significantly advanced by more specific and targeted genetic manipulation. The possibilities of functional genomics, genome editing, and further work in model organisms are almost endless. Coupled with the impact of new imaging modalities (Liu et al. 2018) with higher spatial and temporal resolution in vivo and in vitro makes for exciting possibilities in this field. Such rich data sets also hold further potential to integrate computational modelling and machine learning to better understand the diversity, adaptability, and necessity of specific components within this pathway in different contexts. While efforts at mathematical modelling have been made (Heinzer et al. 2008), clearly there is much more to understand in terms of the geometry, organization, and regulation of COPII-dependent budding. While the Nobel Prize has been awarded and the core machinery defined, still we have ample opportunity to further our understanding of the biology, as well as to translate our current knowledge to derive real impact on human and animal health.
